# Perivascular Adipose-Derived Exosomes Reduce Foam Cell Formation by Regulating Expression of Cholesterol Transporters

**DOI:** 10.3389/fcvm.2021.697510

**Published:** 2021-08-19

**Authors:** Yan Liu, Yan Sun, Xuze Lin, Dai Zhang, Chengping Hu, Jinxing Liu, Yong Zhu, Ang Gao, Hongya Han, Meng Chai, Jianwei Zhang, Yujie Zhou, Yingxin Zhao

**Affiliations:** ^1^Department of Cardiology, Beijing Anzhen Hospital, Capital Medical University, Beijing Institute of Heart Lung and Blood Vessel Disease, Beijing, China; ^2^Department of Cardiology, Fuwai Hospital, State Key Laboratory of Cardiovascular Disease, National Center for Cardiovascular Diseases, Chinese Academy of Medical Sciences and Peking Union Medical College, Beijing, China

**Keywords:** cholesterol transport proteins, cholesterol, perivascular adipose tissue, exosome, macrophage foam cell formation

## Abstract

**Background:** Accumulating evidence demonstrates that perivascular adipose tissue (PVAT) plays an important role in maintaining vascular homeostasis. The formation of macrophage foam cells is a central feature of atherosclerosis. This study aimed to evaluate the effect of PVAT-derived exosomes (EXOs) on the lipid accumulation of macrophages and verify the anti-atherogenic characteristics of PVAT.

**Methods and Results:** We extracted EXOs from the PVAT and subcutaneous adipose tissue (SCAT) of wild-type C57BL/6J mice. After coincubation, the EXOs were taken up by RAW264.7 cells. Oil Red O staining revealed that macrophage foam cell formation and intracellular lipid accumulation were ameliorated by PVAT-EXOs. Flow cytometry showed that PVAT-EXOs significantly reduced macrophage uptake of fluorescence-labelled oxidised low-density lipoprotein (ox-LDL). In addition, high-density lipoprotein-induced cholesterol efflux was promoted by PVAT-EXOs. Western blot analysis showed the downregulation of macrophage scavenger receptor A and the upregulation of ATP-binding cassette transporter A1 and ATP-binding cassette transporter G1, which could be mediated by the overexpression of peroxisome proliferator-activated receptor γ and was independent of liver X receptor α.

**Conclusion:** Our findings suggest that PVAT-EXOs reduce macrophage foam cell formation by regulating the expression of cholesterol transport proteins, which provides a novel mechanism by which PVAT protects the vasculature from atherosclerosis.

## Introduction

Atherosclerosis is a fibrofatty lesion of the artery wall that contributes to stroke, coronary artery disease (CAD), and disabling peripheral artery disease and causes high morbidity and mortality worldwide (1, 2). The formation of macrophage foam cells plays a crucial role in the pathogenesis of atherosclerosis. The lipid homeostasis of macrophages depends on the dynamic balance of cholesterol uptake, efflux and endogenous synthesis. Excessive oxidised low-density lipoprotein (ox-LDL) uptake mediated by macrophage scavenger receptor A (SR-A), the class B scavenger receptor CD36 (3), low-density lipoprotein receptor (LDL-R) and lectin-like ox-LDL receptor-1 (LOX-1), as well as reduced cholesterol efflux via ATP-binding cassette transporter A1 (ABCA1) or ATP-binding cassette transporter G1 (ABGA1) (4) contributes to lipid accumulation.

Perivascular adipose tissue (PVAT) has been shown to have numerous paracrine and endocrine functions and releases a variety of adipocytokines and chemokines (5). Given the adjacent vessel wall, PVAT could have a significant impact on the pathogenesis of atherosclerosis (6). Ren et al. (7) demonstrated that compared with the transplantation of thoracic PVAT from ApoE-/- mice, the transplantation of PVAT from wild-type mice significantly reduced plaque macrophage levels and the messenger RNA expression of inflammatory cytokines. In addition, Terada et al. (8) showed that the transplantation of thoracic PVAT induces the TGF-β1-mediated anti-inflammatory response, which exerts an anti-atherogenic effect. Based on these studies, it can be concluded that under physiological conditions, PVAT possesses anti-inflammatory characteristics and inhibits the development of atherosclerosis. By contrast, under pathological conditions, such as obesity and diabetes, PVAT becomes dysfunctional and secrets pro-inflammatory adipokines that induce endothelial dysfunction and inflammatory cell infiltration, thus contributing to atherosclerosis (9, 10). In this study, we focus on the association between healthy PVAT and macrophage foam cell formation.

Adipose tissue is the main source of exosomes, which are extracellular vesicles with sizes ranging from 40 to 160 nm in diameter (11). Exosomes contain multiple biological factors, such as cell-surface proteins, lipids, metabolites, RNA and DNA (11). Through endocrine or paracrine pathways, exosomes participate in intercellular communication, thereby regulating numerous physiological and pathological processes (12). Flaherty et al. showed that adipocytes release exosomes to modulate macrophage differentiation and function (13). Moreover, Xie et al. suggested that exosomes from the visceral adipose tissue of obese mice regulate macrophage foam cell transformation and polarisation, which promotes the progression of atherosclerosis (14). In this study, we investigated the role of PVAT-derived exosomes in modulating macrophage foam cell formation.

## Materials and Methods

### Isolation of Adipose Tissue-Derived Exosomes

Twelve-week-old male C57BL/6J mice were purchased from the Animal Centre of Capital Medical University (Beijing, China). The mice were fed a standard diet. The procedure was approved by the Animal Care and Use Committee of Capital Medical University. The mice were anaesthetized intraperitoneally. PVAT of aorta and inguinal subcutaneous adipose tissue (SCAT) were harvested and washed with phosphate-buffered saline (PBS). The tissue was minced into small pieces (1 mm^3^) and cultured in DMEM-F12 medium supplemented with 100 U/ml penicillin-streptomycin at 5% CO_2_ and 37°C for 24 h. The media was collected and centrifuged at 3,000 g for 10 min to remove the cells. The supernatant was then filtered through a 0.22 μm syringe-driven philtre to remove any remaining cellular debris. The exosomes were isolated through ultracentrifugation and resuspended in PBS. After measurement of concentration with BCA Protein Assay Kit (Beyotime, Shanghai, China), the isolated exosomes were diluted to 1 mg/ml and stored at −80°C until use.

### Identification of Exosomes

Exosomes were visualised with transmission electron microscopy (TEM; JEM-1220, Jeol, Tokyo, Japan). In addition, nanoparticle tracking analysis (NTA; Malvern Instruments, Malvern, UK) was performed to estimate the size distribution of the exosomes. For further identification, specific exosome markers were analysed by western blotting, including CD9, CD63 and TSG101.

### Exosome Trafficking Assay

RAW264.7 cells were cultured in RPMI 1640 medium supplemented with 10% foetal bovine serum (FBS) and 100 U/ml penicillin-streptomycin at 5% CO_2_ and 37°C. The exosomes were labelled with PKH26 Red Fluorescent Cell Linker Kit (Sigma-Aldrich, MO, USA) according to the manufacturer's protocol, and washed 5 times with PBS. The last PBS supernatant collected after exosome labelling was used as control. The cells were incubated with exosomes (10 μg/ml) for 12 h. After being washed with PBS, the cytoskeleton was stained with phalloidin (YEASEN Biotech, Shanghai, China) and the nucleus with 4',6-diamidino-2-phenylindole (DAPI; Invitrogen, Carlsbad, CA). Fluorescence was analysed by a LEICA TCS-SP2 laser confocal microscope (Leica Microsystems, Wetzlar, Germany).

### Flow Cytometry

RAW264.7 cells were incubated in RPMI 1640 medium with 10 μg/ml SCAT-EXOs, 10 μg/ml PVAT-EXOs or equal amounts of PBS for 24 h, and then 50 ng/ml lipopolysaccharide (LPS, Sigma-Aldrich) was added for another 24 h. After being washed twice with cold PBS, the cells were harvested and incubated for 30 min at 4°C in the dark with antibodies including APC-anti-mouse F4/80 (Biolegend, CA, USA), PE-anti-mouse CD80 (Biolegend) and FITC-anti-mouse CD206 (Biolegend). The cells were analysed by flow cytometry (BD Pharmingen, NJ, USA) and FlowJo 7.5 (FlowJo, OR, USA).

### Enzyme Linked Immunosorbent Assay

RAW264.7 cells were pretreated with or without EXOs and stimulated with LPS as described for flow cytometry. The supernatants were collected after centrifugation, and the concentrations of TNF-a and IL-6 were measured according to manual of Mouse ELISA Kit (Dakewe Biotech, Shenzhen, China).

### Oil Red O Staining and Cholesterol Quantification

RAW264.7 cells were incubated in RPMI 1640 medium containing 0.5% bovine serum albumin (BSA) and 100 U/ml penicillin-streptomycin with or without 10 μg/ml exosomes for 12 h. Then, the cells were treated with 50 μg/ml ox-LDL (Yiyuan Biotech, Guangzhou, China) for an additional 48 h. After being washed with PBS three times, the cells were fixed with 4% paraformaldehyde for 20 min and dehydrated with 60% isopropanol. The cells were stained with filtered Oil Red O solution (Solarbio, Beijing, China) for 10–20 min, and the cell nuclei were counterstained with Mayer's haematoxylin (Solarbio) for 1–2 min. Images were captured with light microscopy. The total cholesterol and free cholesterol levels were measured by assay kits (Solarbio) according to the manufacturer's instructions. The level of cholesterol esters was obtained as total cholesterol minus free cholesterol. In addition, the ultrastructure of foam cells was detected with TEM.

### Cholesterol Uptake Assay

RAW264.7 cells were cultured in RPMI 1640 medium supplemented with 0.5% BSA and 100 U/ml penicillin-streptomycin with or without 10 μg/ml exosomes for 2 h. Then, 10 μg/ml fluorescence-labelled ox-LDL (Dil-oxLDL; Yiyuan Biotech) was added to each group and incubated for an additional 4 h. After being washed three times, the cells were resuspended in 0.5 ml of PBS. The Dil-oxLDL uptake was visualised with a fluorescence microscope and further analysed by flow cytometry.

### Cholesterol Efflux Assay

RAW264.7 cells were cultured in RPMI 1640 medium containing 0.2% BSA, 1% penicillin-streptomycin, and 1 μg/ml 3-hexanoyl-NBD cholesterol (Cayman Chemical, MI, USA) for 24 h. After being washed with PBS, the cells were cultured in medium with or without 10 μg/ml exosomes for 2 h. Cholesterol efflux was stimulated by 50 μg/ml high-density lipoprotein (HDL; Peking Union-Biology, Beijing, China). The supernatant and cell lysates were collected and transferred to 96-well plates. The fluorescence was measured with a microplate reader (Varian Australia, VIC, Australia) at excitation/emission maxima of 473/536 nm. Cholesterol efflux was calculated by the following formula: Media fluorescence intensity/(Cell fluorescence intensity + Media fluorescence intensity) × 100%.

### Western Blotting

The proteins from exosomes and cells were extracted with RIPA lysis buffer (Solarbio) supplemented with proteinase inhibitors (Solarbio). Each well was loaded with 30 μg of protein. The proteins were transferred to polyvinylidene fluoride membranes (Millipore, Schwalbach, Germany), and the membranes were blocked with 5% skimmed milk for 1 h. Then, the membranes were probed with primary antibodies (1:1,000) overnight at 4°C. Antibodies against CD9, CD63, TSG101, SR-A, CD36, LDL-R, LOX-1, ABCA1, ABCG1, liver X receptor α (LXRα), and peroxisome proliferator-activated receptor γ (PPARγ) were purchased from Abcam (Cambridge, UK). β-actin (Cell Signalling Technology) was used as the loading control. In addition, the membranes were incubated with the corresponding HRP-conjugated secondary antibodies in the dark at room temperature for 1 h. The immunoreactivity was visualised with a ChemiDoc MP Imaging System (Bio-Rad) and analysed with ImageJ software (MA, USA).

### Real-Time Polymerase Chain Reaction

Total RNA was extracted from exosomes or macrophages with TRIzol reagent (Invitrogen). The isolated RNA was reverse transcribed using EasyScript All-in-One First-Strand cDNA Synthesis SuperMix for qPCR (Transgen Biotech, Beijing, China). Quantitative PCR was performed on a Bio-Rad iQ5 Real-Time PCR Detection System with PerfectStart Green qPCR SuperMix (Transgen Biotech, Beijing, China). The following primers were used: CD36, forward 5′- GGACATTGAGATTCTTTTCCTCTG-3′ and reverse 5′- GCAAAGGCATTGGCTGGAAGAAC-3′; SR-A, forward 5′- CTGAGACCTCTGGAACAGGCAT-3′ and reverse 5′- TGCACTAGCAGTGCCATCCTCT-3′; LDL-R, forward 5′- GAATCTACTGGTCCGACCTGTC-3′ and reverse 5′- CTGTCCAGTAGATGTTGCGGTG-3′; LOX-1, forward 5′- GTCATCCTCTGCCTGGTGTTGT-3′ and reverse 5′- TGCCTTCTGCTGGGCTAACATC-3′; ABCA1, forward 5′- GGAGCCTTTGTGGAACTCTTCC-3′ and reverse 5′- CGCTCTCTTCAGCCACTTTGAG-3′; and ABCG1, forward 5′- GACACCGATGTGAACCCGTTTC-3′ and reverse 5′- GCATGATGCTGAGGAAGGTCCT-3′. The gene expression level was normalised to that of glyceraldehyde 3-phosphate dehydrogenase (GAPDH). All samples were repeated three times, and the data were calculated using the 2- ΔΔCt method.

### Statistical Analysis

Statistical analyses were conducted using GraphPad Prism 8.2.1 (GraphPad Software, CA, USA). The data are presented as the mean ± standard deviation. One-way analysis of variance (ANOVA) with Tukey's *post-hoc* adjustment was performed to identify significant differences among groups. P-values < 0.05 were considered statistically significant.

## Results

### Characterisation and Endocytosis of EXOs

**Figure 1:**
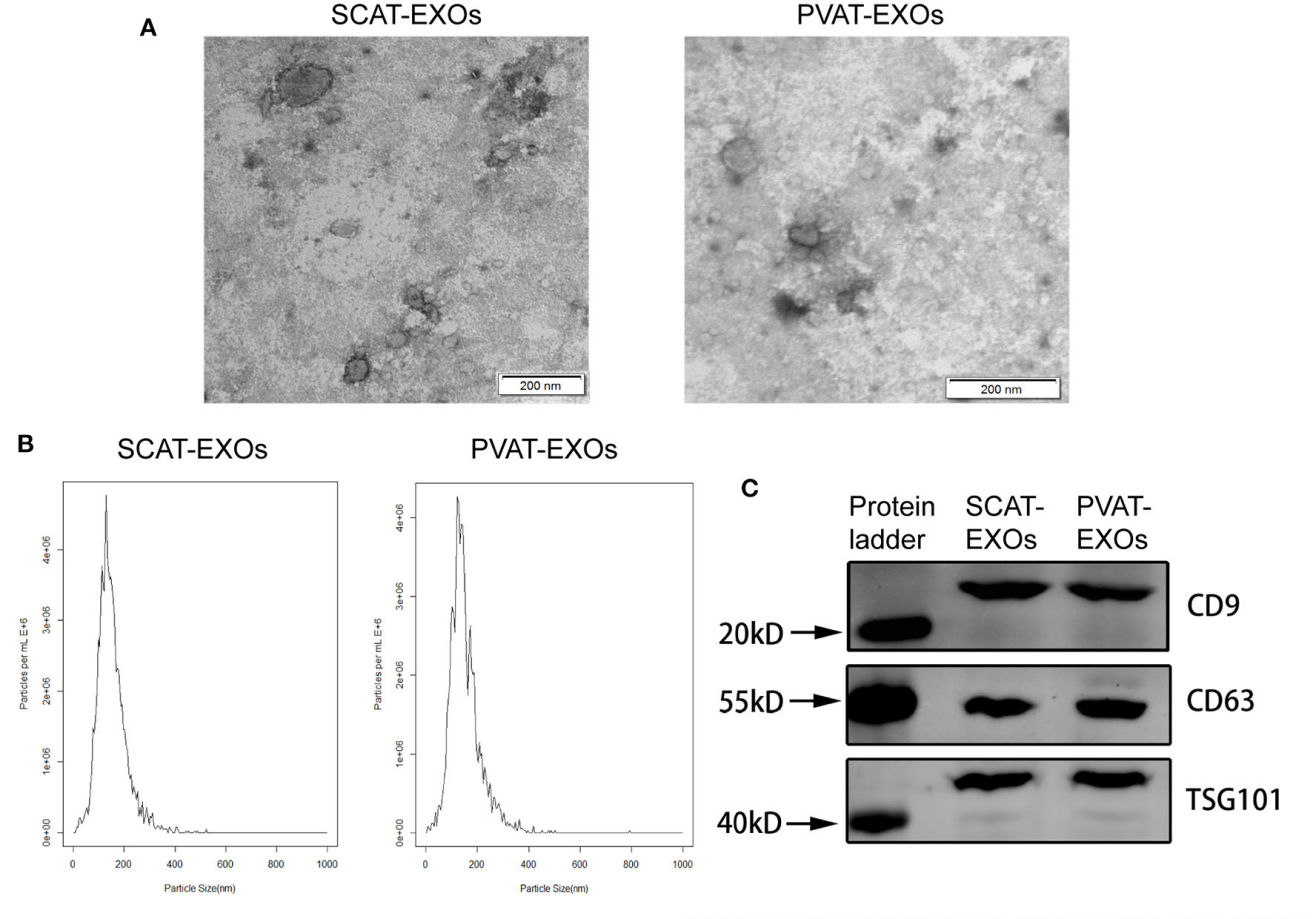
Characterisation of SCAT-EXOs and PVAT-EXOs. Exosomes were identified by transmission electron microscopy **(A)**. Scale bars: 200 nm. Nanoparticle tracking analysis showed the particle size distribution of exosomes **(B)**. The exosome-related protein markers CD9, CD63, and TSG101 were measured by Western blotting **(C)**.

**Figure 2:**
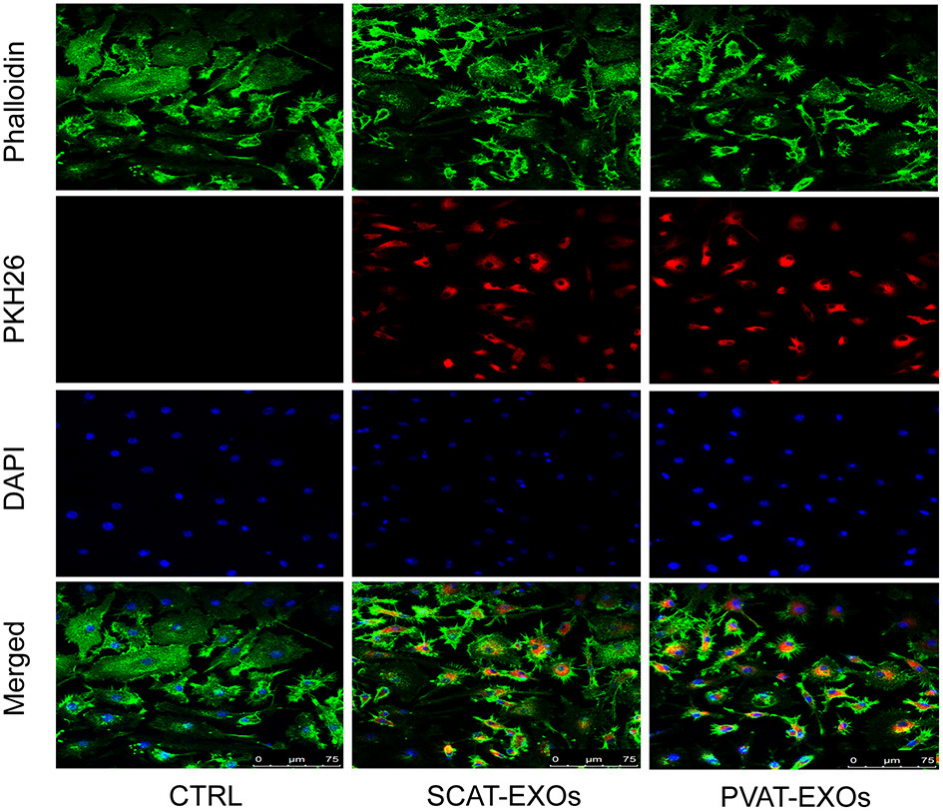
SCAT-EXOs and PVAT-EXOs were transferred into macrophages. Exosomes were labelled with PKH26 (red) and incubated with RAW264.7 cells for 12 h. The cells were then stained with phalloidin (green) and DAPI (blue). Fluorescence signals were analysed by laser confocal microscopy. Ctrl: control group. Scale bars: 75 μm.

We performed three assays to identify the characteristics of the EXOs. First, the EXOs were verified by TEM as cup- or sphere-shaped in morphology (Figure 1A). In addition, NTA identified EXOs 50–150 nm in diameter (Figure 1B). Moreover, western blot analysis revealed the presence of the EXO-related protein markers CD9, CD63, and TSG101 (Figure 1C). We labelled exosomes with PKH26 and incubated the exosomes with macrophages for 12 h. Under a laser confocal microscope, we verified that both SCAT-EXOs and PVAT-EXOs were taken up by macrophages (Figure 2).

### PVAT-EXOs Reduce the Formation of Macrophage Foam Cells

**Figure 3:**
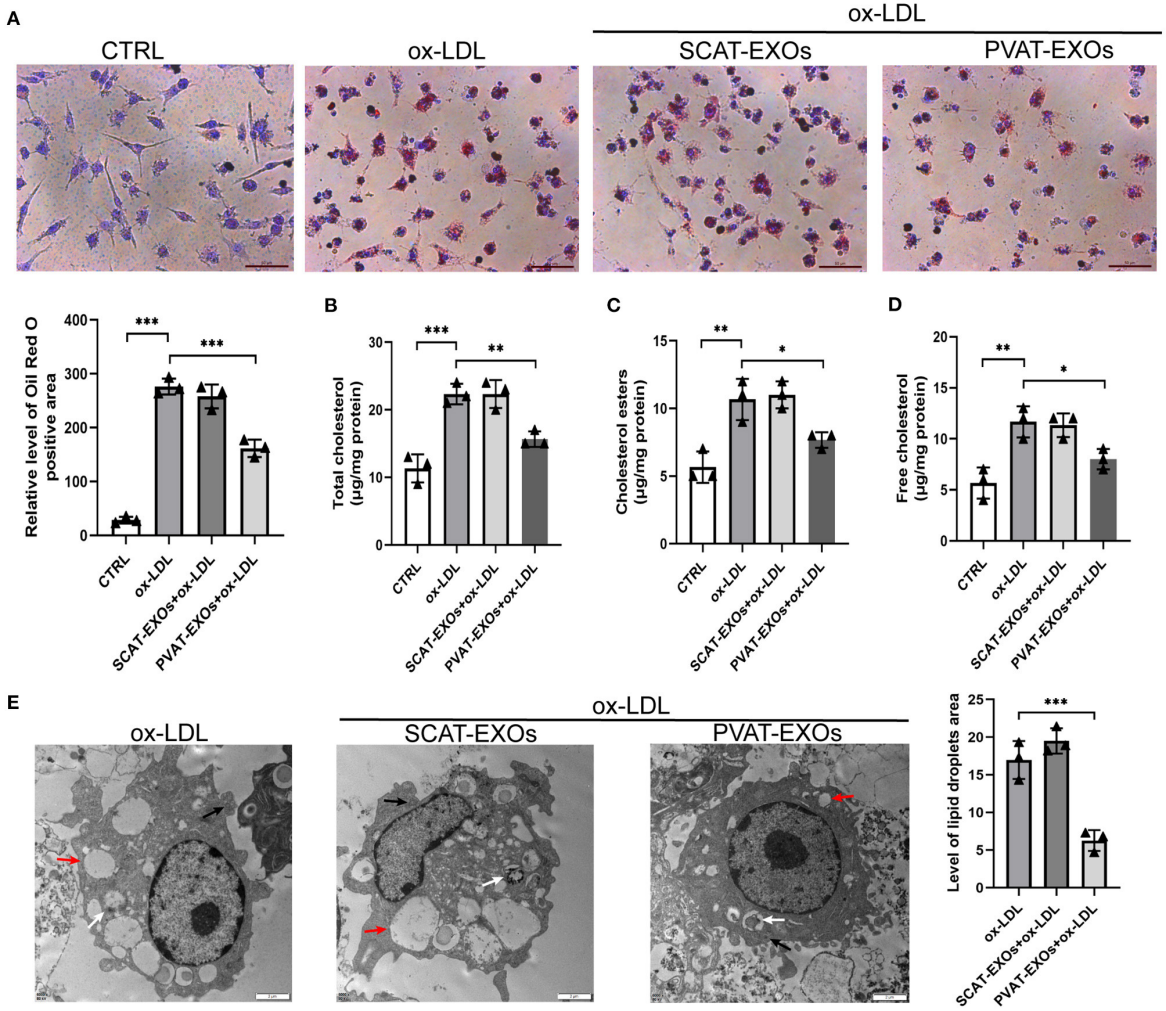
PVAT-EXOs reduced macrophage foam cell formation. RAW264.7 cells were incubated with SCAT-EXOs, PVAT-EXOs or equal amounts of PBS and then incubated with or without ox-LDL. Oil Red O staining indicated that PVAT-EXOs reduced macrophage foam cell formation **(A)**. Scale bars: 50 μm. Lipid accumulation in macrophages was confirmed by determining the levels of total cholesterol **(B)**, cholesterol esters **(C)** and free cholesterol **(D)**. Ctrl: control group. The ultrastructure of foam cells was detected with transmission electron microscopy **(E)**. Black arrows indicate phagosomes, white arrows indicate lysosomes, and red arrows indicate lipid droplets. Scale bars: 2 μm. *P < 0.05, **P < 0.01, ***P < 0.001 vs. the ox-LDL treated group.

**Figure 4:**
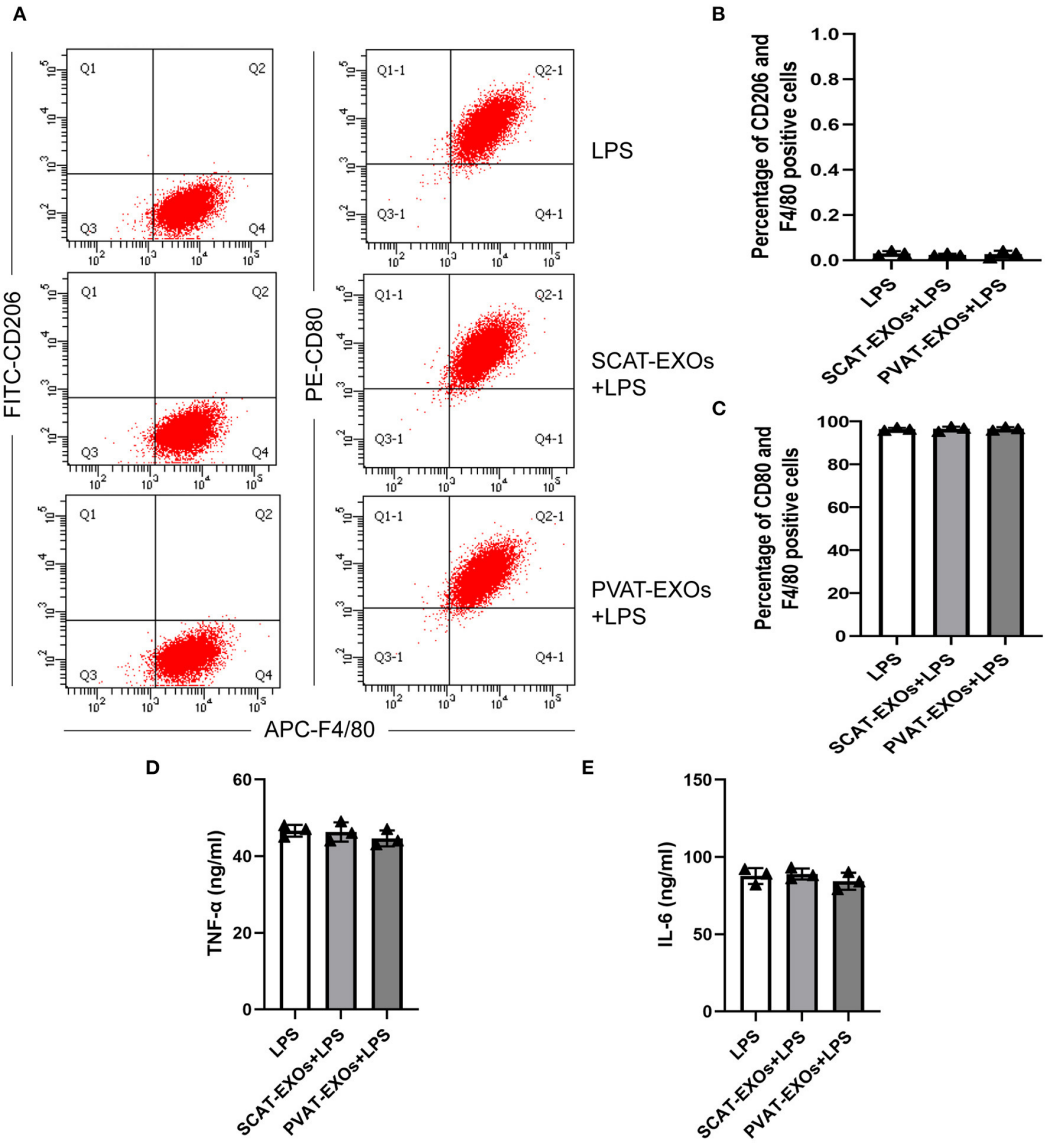
Macrophage polarisation was not affected by SCAT-EXOs or PVAT-EXOs. RAW264.7 cells were incubated with SCAT-EXOs, PVAT-EXOs or equal amounts of PBS and then incubated with lipopolysaccharide (LPS). The phenotypes of cells were analysed by flow cytometry **(A)**. There was no significant difference in the percentages of CD206+/F4/80+ cells or CD80+/F4/80+ cells between the groups **(B,C)**. The production of TNF-a **(D)** or IL-6 **(E)** was not affected by EXOs.

To verify the effect of PVAT-EXOs on macrophage foam cells, we cultured macrophages with EXOs and treated the cells with ox-LDL. Oil Red O staining showed that SCAT-EXOs had no significant effect on macrophage foam cell formation. Conversely, compared with that in the ox-LDL group, lipid accumulation was significantly reduced by PVAT-EXO pretreatment (Figure 3A). To further determine the level of intracellular cholesterol, we measured the total and free cholesterol in each group and calculated cholesterol esters by subtraction. Compared with those of the ox-LDL group, PVAT-EXO incubation significantly reduced total cholesterol (Figure 3B), cholesterol esters (Figure 3C) and free cholesterol (Figure 3D) in macrophages. In addition, there was no significant difference between the groups with or without SCAT-EXOs. As shown in Figure 3E, the ultrastructure of foam cells was observed by TEM, including phagosomes, lysosomes and lipid droplets. Compared with macrophages treated with ox-LDL, PVAT-EXOs significantly reduced the amount of lipid droplets, while it was not significantly changed by SCAT-EXOs. Furthermore, the flow cytometry and ELISA did not show an influence of exosomes on the phenotypic transition and cytokine production of macrophages (Figure 4).

### PVAT-EXOs Regulate Cholesterol Uptake and Efflux in Macrophages

**Figure 5:**
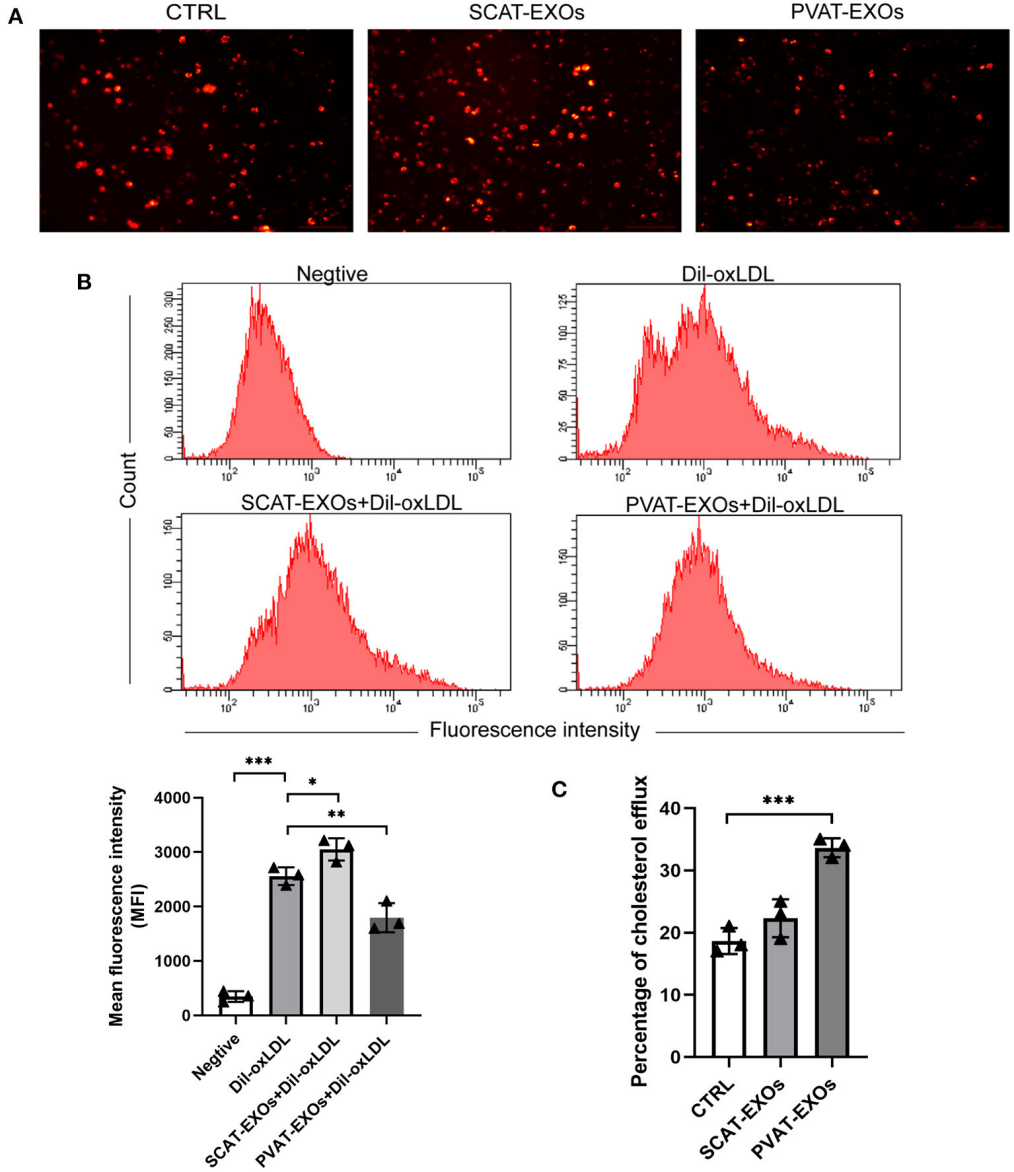
PVAT-EXOs reduced Dil-oxLDL uptake and promote cholesterol efflux in macrophages. After treatment with SCAT-EXOs, PVAT-EXOs or PBS, Dil-oxLDL uptake by RAW264.7 cells was analysed by fluorescence microscopy **(A)**. In addition, the fluorescence intensities were quantified by flow cytometry **(B)**. HDL-mediated cholesterol efflux was significantly promoted by PVAT-EXOs **(C)**. Ctrl: control group. *P < 0.05, **P < 0.01, ***P < 0.001 vs. the Dil-oxLDL group or control group.

We incubated macrophages with PBS, SCAT-EXOs and PVAT-EXOs for 12 h and then treated the cells with Dil-oxLDL for 6 h. As shown in Figure 5A, compared with that in the Dil-oxLDL group, there was a substantial reduction in fluorescence in the PVAT-EXO group. We further quantified the flow cytometry results, which revealed that the cholesterol uptake by macrophages was significantly diminished by PVAT-EXOs and was significantly increased by SCAT-EXOs (Figure 5B). Additionally, the cholesterol efflux of macrophages was measured via the fluorescence intensity of NBD cholesterol. We found that PVAT-EXOs significantly promoted cholesterol efflux by macrophages, while the fluorescence intensity in the SCAT-EXO group was not distinctly different from that of the control group (Figure 5C).

### PVAT-EXOs Alter the Expression of Cholesterol Transporters in Macrophages

**Figure 6:**
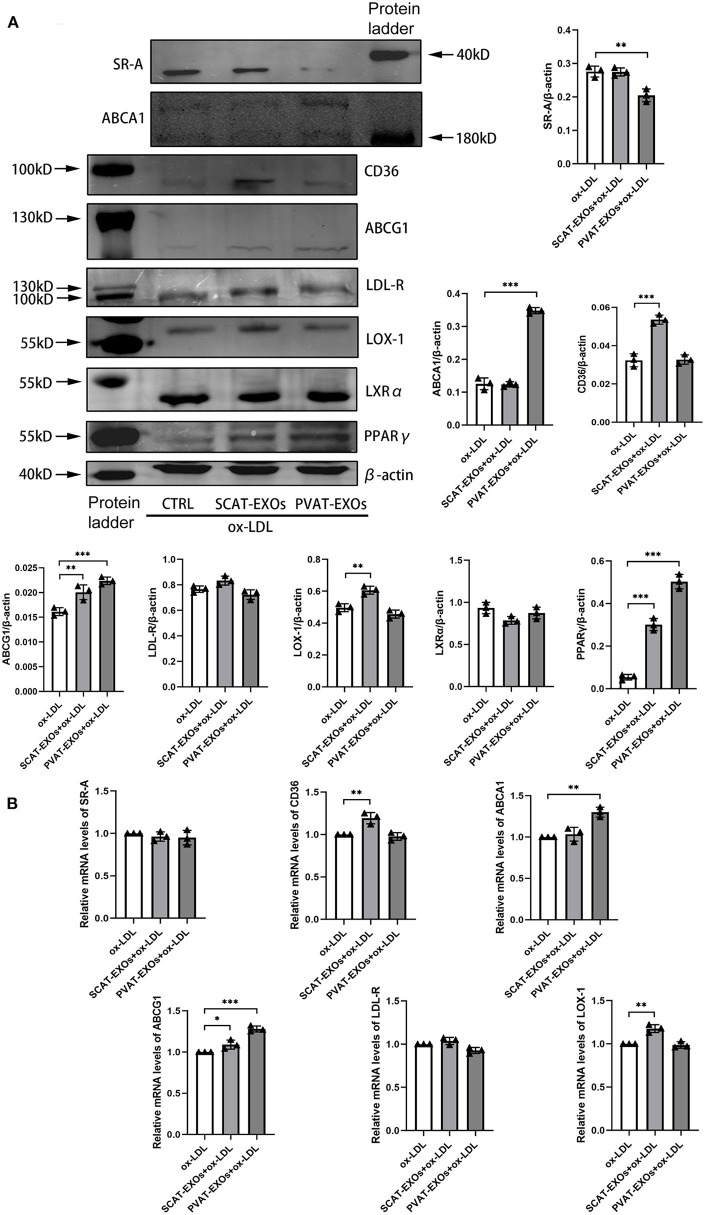
The expression of cholesterol transporters in macrophages was regulated by SCAT-EXOs and PVAT-EXOs. RAW264.7 cells were incubated with SCAT-EXOs, PVAT-EXOs or equal amounts of PBS and then incubated with ox-LDL. Western blot analysis showed the expression of the cholesterol transport proteins SR-A, CD36, LDL-R, LOX-1, ABCA1, and ABCG1 and the upstream proteins LXRα and PPARγ **(A)**. The expression levels were normalised to β-actin. The mRNA expression levels of SR-A, CD36, LDL-R, LOX-1, ABCA1 and ABCG1 were analysed by real-time PCR and normalised to GAPDH **(B)**. Ctrl: control group. *P < 0.05, **P < 0.01, ***P < 0.001 vs. the ox-LDL group.

SR-A, CD36, LDL-R, and LOX-1 are cholesterol uptake transporters, while ABCA1 and ABCG1 mediate cholesterol efflux. To explore the effects of EXOs on cholesterol transporter expression, we incubated macrophages with EXOs for 24 h and treated the cells with ox-LDL for another 6 h. Our results showed that SR-A expression was significantly reduced by PVAT-EXOs, while CD36 and LOX-1 expression were significantly increased by SCAT-EXOs (Figure 6A). Both ABCA1 and ABCG1 were upregulated by PVAT-EXOs. There was no significant difference of LDL-R between each group. Furthermore, PPARγ/LXRα are upstream regulatory proteins of ABCA1 and ABCG1. We found that the expression of PPARγ was enhanced by PVAT-EXOs and SCAT-EXOs, and the change in the former was more significant than that of the latter. In contrast, LXRα did not differ after EXO stimulation. The transcriptional levels of the corresponding mRNAs were confirmed by RT-PCR (Figure 6B). PVAT-EXOs significantly enhanced the mRNA expression of ABCA1 and ABCG1. CD36, ABCG1, and LOX-1 mRNA levels were increased by SCAT-EXOs.

## Discussion

In the current study, we found that PVAT-derived exosomes significantly downregulated the expression of the cholesterol influx transporter SR-A and upregulated the expression of the cholesterol efflux transporters ABCA1 and ABCG1, thereby reducing cholesterol accumulation in macrophages and the formation of foam cells.

In human, visceral adipose tissue, SCAT and most PVAT are white adipose tissue, which stores energy, secrets adipokines and vasoactive factors and causes cardiometabolic disorders after excess accumulation (15). While in mice, SCAT is beige adipose tissue, which could be induced to brown adipose tissue, and is related with thermogenesis, anti-inflammatory properties and cardioprotective effect (15). The differences between PVAT and SCAT have been addressed. Compared with pericarotid adipose tissue, the MCP-1 gene expression was significantly higher in SCAT, while in patients with carotid stenosis the expression was lower in SCAT (16). The pro-inflammatory mediator IL-6, TNF-α, MCP-1 and adiponectin were found more abundant in SCAT vs. PVAT (17), suggesting the pro-inflammatory phenotype is attenuated in PVAT. In recent years, accumulating evidence suggests that PVAT is a novel factor that modulates vascular biology (18, 19). PVAT releases a wide range of biologically active molecules that participate in inflammatory responses (20, 21), endothelial function (22) and vascular smooth muscle proliferation (23). Under physiological conditions, PVAT was shown to protect the vasculature from atherosclerosis. Adiponectin is an important adipokine that participates in anti-atherogenic processes (24). Margaritis et al. reported that adiponectin regulated eNOS coupling to improve the redox state in vessels, and adiponectin gene expression in PVAT was upregulated by peroxidation products via a PPARγ-dependent mechanism (25). By promoting eNOS phosphorylation and reducing PVAT inflammation, adiponectin improves endothelial-dependent vascular relaxation (22). In addition, omentin was shown to negatively regulate atherosclerosis development by modulating foam cell formation and the inflammatory response in macrophages (26). Consistent with previous studies, we verified that PVAT-derived exosomes were taken up by macrophages and acted as protective factors against foam cell formation. Research on PVAT-derived exosomes is quite rare. Li et al. showed that extracellular vesicles from obese mouse PVAT were taken up by neighbouring smooth muscle cells, leading to phenotypic switching and arterial remodelling (27). Our study is the first to reveal the adversarial association between PVAT-EXOs and the formation of macrophage foam cells. Considering its anatomical location, PVAT could play a fundamental role in the development of atherosclerosis.

Studies on exosomes from different types of adipose tissue and their effects on macrophages have been published recently. Wei et al. indicated that a high-fat diet changed the miRNA profile of mouse visceral adipose tissue-derived exosomes into a proinflammatory phenotype that promoted M1 macrophage polarisation (28). Similarly, Pan et al. showed that miR-34a in epididymal white adipose tissue-derived exosomes was positively associated with obesity and drove the polarisation of macrophages towards the M1 phenotype (29). In contrast, in diet-induced obese mice, inflammation reduction, alternatively activated M2 macrophage polarisation, and the beiging of white adipose tissue were facilitated by exosomes from adipose-derived stem cells (30). However, in this study, we failed to identify the effect of wild-type mouse PVAT-EXOs on M1 macrophage polarisation and inflammatory cytokine production. The high-fat diet-related characteristics of PVAT, which may be proinflammatory, warrant further exploration. Xie et al. reported that exosomes from visceral adipose tissue significantly promoted macrophage foam cell transformation via the suppression of ABCA1- or ABCG1-mediated cholesterol efflux but not SCAT-EXOs (14). Consistently, SCAT-EXOs did not affect the lipid accumulation of macrophages in our study. Furthermore, PVAT-EXOs were shown to markedly reduce the generation of foam cells through the downregulation of SR-A and upregulation of cholesterol efflux transporters, which was accompanied by exacerbated PPARγ expression.

LXRα and PPARγ are ligand-activated nuclear receptors that regulate lipid metabolism in macrophages (31). Moore et al. indicated that PPARγ regulates SR-A expression through posttranscriptional mechanisms, as PPARγ activation leads to a reduction in SR-A protein without altering SR-A mRNA expression (32). Consistent with the previous study, our data revealed a significant reduction in SR-A protein in PVAT-EXO-treated macrophages, which may be mediated by an increase in PPARγ. On the other hand, the expressions of cholesterol uptake protein CD 36, LDL-R, and LOX-1 were not significantly changed by PVAT-EXOs, suggesting the cholesterol uptake decrease should be attributed to SR-A. Activation of the PPARγ-LXRα-ABCA1/ABCG1 pathway has been shown to enhance cholesterol efflux (33). Claudel et al. revealed that PPARγ agonists and LXR/retinoid X receptor ligands induce ABC-1-mediated cholesterol efflux, which significantly reduces atherosclerotic lesions in ApoE–/– mice (34). In addition, Ruan et al. suggested a similarity between PPAR and LXRα in regulating the expression of ABCA1 and the presence of an additive effect when used together (35). In the present study, we suggested that PVAT-EXOs induce increased expression of PPARγ, which accompanied with the upregulation of ABCA1 and ABCG1, without a significant change in LXRα. Earlier research provided similar results. Li et al. demonstrated that a PPARγ agonist promoted ABCG1 expression in LXR double-knockout and wild-type macrophages (36). Additionally, a PPARγ agonist significantly enhanced the expression of ABCG1 in hypercholesterolaemic LDLR–/– mice (36). Therefore, these results suggest that PVAT-EXOs induce cholesterol efflux via the PPARγ-ABCA1/ABCG1 pathway, which is independent of LXRα.

## Conclusions

Our results suggest that PVAT-derived exosomes reduce the formation of macrophage foam cells, which could be protective factors against atherosclerosis. PVAT-derived exosomes downregulate the expression of SR-A to reduce cholesterol uptake in macrophages. In addition, the overexpression of ABCA1 and ABCG1 was induced to promote cholesterol efflux. This anti-atherogenic effect might be mediated by upstream regulation of PPARγ. The use of PVAT-derived exosomes as a promising prevention and therapeutic strategy for atherosclerosis warrants further investigation.

## Data Availability Statement

The original contributions presented in the study are included in the article/supplementary material, further inquiries can be directed to the corresponding author/s.

## Ethics Statement

The animal study was reviewed and approved by The Animal Care and Use Committee of Capital Medical University.

## Author Contributions

YL performed this study, collected data, drafted this article including drawing figures and revising the article. YS and XL helped to perform the study, and revised the article. DZ, CH, JL, YZ, and AG collected and analysed part of data and made revisions. HH, MC, JZ, YZho, and YZha made adjustments to the experimental design and revised the figures. All authors read and approved the final version of the article.

## Conflict of Interest

The authors declare that the research was conducted in the absence of any commercial or financial relationships that could be construed as a potential conflict of interest.

## Publisher's Note

All claims expressed in this article are solely those of the authors and do not necessarily represent those of their affiliated organizations, or those of the publisher, the editors and the reviewers. Any product that may be evaluated in this article, or claim that may be made by its manufacturer, is not guaranteed or endorsed by the publisher.

## Abbreviations

PVAT: perivascular adipose tissue
EXOs: exosomes
SCAT: subcutaneous adipose tissue
ox-LDL: oxidised low-density lipoprotein
CAD: coronary artery disease
SR-A: scavenger receptor A
LDL-R: low-density lipoprotein receptor
LOX-1: lectin-like ox-LDL receptor-1
ABCA1: ATP-binding cassette transporter A1
ABGA1: ATP-binding cassette transporter G1
PBS: phosphate-buffered saline
TEM: transmission electron microscopy
NTA: nanoparticle tracking analysis
FBS: foetal bovine serum
DAPI: 4', 6-diamidino-2-phenylindole
BSA: bovine serum albumin
Dil-oxLDL: fluorescence-labelled ox-LDL
HDL: high-density lipoprotein
LXRα: liver X receptor α
PPARγ: peroxisome proliferator-activated receptor γ
GAPDH: glyceraldehyde 3-phosphate dehydrogenase.

## References

[1] Libby P Buring JE Badimon L Hansson GK Deanfield J Bittencourt MS. Atherosclerosis. Nat Rev Dis Primers. (2019) 5:56. 10.1038/s41572-019-0106-z 31420554

[2] Virani SS Alonso A Benjamin EJ Bittencourt MS Callaway CW Carson AP. Heart disease and stroke statistics-2020 update: a report from the American Heart Association. Circulation. (2020) 141:e139–96. 10.1161/CIR.0000000000000757 31992061

[3] Mäkinen PI Lappalainen JP Heinonen SE Leppänen P Lähteenvuo MT Aarnio JV. Silencing of either SR-A or CD36 reduces atherosclerosis in hyperlipidaemic mice and reveals reciprocal upregulation of these receptors. Cardiovasc Res. (2010) 88:530–8. 10.1093/cvr/cvq235 20634212

[4] Castaño D Rattanasopa C Monteiro-Cardoso VF Corlianò M Liu Y Zhong S. Lipid efflux mechanisms, relation to disease and potential therapeutic aspects. Adv Drug Deliv Rev. (2020) 159:54–93. 10.1016/j.addr.2020.04.013 32423566

[5] Szasz T Webb RC. Perivascular adipose tissue: more than just structural support. Clin Sci. (2012) 122:1–12. 10.1042/CS20110151 21910690 PMC3966487

[6] Ahmadieh S Kim HW Weintraub NL. Potential role of perivascular adipose tissue in modulating atherosclerosis. Clin Sci. (2020) 134:3–13. 10.1042/CS20190577 31898749 PMC6944729

[7] Ren L Wang L You T Liu Y Wu F Zhu L. Perivascular adipose tissue modulates carotid plaque formation induced by disturbed flow in mice. J Vasc Surg. (2019) 70:927–36.e924. 10.1016/j.jvs.2018.09.064 30777689

[8] Terada K Yamada H Kikai M Wakana N Yamamoto K Wada N. Transplantation of periaortic adipose tissue inhibits atherosclerosis in apoE(-/-) mice by evoking TGF-β1-mediated anti-inflammatory response in transplanted graft. Biochem Biophys Res Commun. (2018) 501:145–51. 10.1016/j.bbrc.2018.04.196 29705699

[9] Liu Y Sun Y Hu C Liu J Gao A Han H. Perivascular adipose tissue as an indication, contributor to, and therapeutic target for atherosclerosis. Front Physiol. (2020) 11:615503. 10.3389/fphys.2020.615503 33391033 PMC7775482

[10] Qi XY Qu SL Xiong WH Rom O Chang L Jiang ZS. Perivascular adipose tissue (PVAT) in atherosclerosis: a double-edged sword. Cardiovasc Diabetol. (2018) 17:134. 10.1186/s12933-018-0777-x 30305178 PMC6180425

[11] Kalluri R LeBleu VS. The biology, function, and biomedical applications of exosomes. Science. (2020) 367:eaau6977. 10.1126/science.aau6977 32029601 PMC7717626

[12] Pegtel DM Gould SJ. Exosomes. Annu Rev Biochem. (2019) 88:487–514. 10.1146/annurev-biochem-013118-111902 31220978

[13] Flaherty SE 3rd Grijalva A Xu X Ables E Nomani A Ferrante AW Jr. A lipase-independent pathway of lipid release and immune modulation by adipocytes. Science. (2019) 363:989–93. 10.1126/science.aaw2586 30819964 PMC6579605

[14] Xie Z Wang X Liu X Du H Sun C Shao X. Adipose-derived exosomes exert proatherogenic effects by regulating macrophage foam cell formation and polarization. J Am Heart Assoc. (2018) 7:e007442. 10.1161/JAHA.117.007442 29502100 PMC5866320

[15] Koenen M Hill MA Cohen P Sowers JR. Obesity, adipose tissue and vascular dysfunction. Circ Res. (2021) 128:951–68. 10.1161/CIRCRESAHA.121.318093 33793327 PMC8026272

[16] Pandzic Jaksic V Grizelj D Livun A Ajduk M Boscic D Vlasic A. Inflammatory gene expression in neck perivascular and subcutaneous adipose tissue in men with carotid stenosis. Angiology. (2021). 10.1177/00033197211012539. [Epub ahead of print]. 33906471

[17] Mauro CR Ilonzo G Nguyen BT Yu P Tao M Gao I. Attenuated adiposopathy in perivascular adipose tissue compared with subcutaneous human adipose tissue. Am J Surg. (2013) 206:241–4. 10.1016/j.amjsurg.2012.07.032 23352378 PMC3688688

[18] Brown NK Zhou Z Zhang J Zeng R Wu J Eitzman DT. Perivascular adipose tissue in vascular function and disease: a review of current research and animal models. Arterioscler Thromb Vasc Biol. (2014) 34:1621–30. 10.1161/ATVBAHA.114.303029 24833795 PMC4104287

[19] Liang X Qi Y Dai F Gu J Yao W. PVAT: an important guardian of the cardiovascular system. Histol Histopathol. (2020) 35:779–87. 10.14670/HH-18-211 32080826

[20] Omar A Chatterjee TK Tang Y Hui DY Weintraub NL. Proinflammatory phenotype of perivascular adipocytes. Arterioscler Thromb Vasc Biol. (2014) 34:1631–6. 10.1161/ATVBAHA.114.303030 24925977 PMC4113719

[21] Nosalski R Guzik TJ. Perivascular adipose tissue inflammation in vascular disease. Br J Pharmacol. (2017) 174:3496–513. 10.1111/bph.13705 28063251 PMC5610164

[22] Sena CM Pereira A Fernandes R Letra L Seica RM. Adiponectin improves endothelial function in mesenteric arteries of rats fed a high-fat diet: role of perivascular adipose tissue. Br J Pharmacol. (2017) 174:3514–26. 10.1111/bph.13756 28236429 PMC5610162

[23] Miao CY Li ZY. The role of perivascular adipose tissue in vascular smooth muscle cell growth. Br J Pharmacol. (2012) 165:643–58. 10.1111/j.1476-5381.2011.01404.x 21470202 PMC3315037

[24] Fasshauer M Blüher M. Adipokines in health and disease. Trends Pharmacol Sci. (2015) 36:461–70. 10.1016/j.tips.2015.04.014 26022934

[25] Margaritis M Antonopoulos AS Digby J Lee R Reilly S Coutinho P. Interactions between vascular wall and perivascular adipose tissue reveal novel roles for adiponectin in the regulation of endothelial nitric oxide synthase function in human vessels. Circulation. (2013) 127:2209–21. 10.1161/CIRCULATIONAHA.112.001133 23625959

[26] Hiramatsu-Ito M Shibata R Ohashi K Uemura Y Kanemura N Kambara T. Omentin attenuates atherosclerotic lesion formation in apolipoprotein E-deficient mice. Cardiovasc Res. (2016) 110:107–17. 10.1093/cvr/cvv282 26714927

[27] Li X Ballantyne LL Yu Y Funk CD. Perivascular adipose tissue-derived extracellular vesicle miR-221-3p mediates vascular remodeling. FASEB J. (2019) 33:12704–22. 10.1096/fj.201901548R 31469602 PMC6902668

[28] Wei M Gao X Liu L Li Z Wan Z Dong Y. Visceral adipose tissue derived exosomes exacerbate colitis severity via pro-inflammatory MiRNAs in high fat diet fed mice. ACS Nano. (2020) 14:5099–110. 10.1021/acsnano.0c01860 32275391

[29] Pan Y Hui X Hoo RLC Ye D Chan CYC Feng T. Adipocyte-secreted exosomal microRNA-34a inhibits M2 macrophage polarization to promote obesity-induced adipose inflammation. J Clin Invest. (2019) 129:834–49. 10.1172/JCI123069 30667374 PMC6355214

[30] Zhao H Shang Q Pan Z Bai Y Li Z Zhang H. Exosomes from adipose-derived stem cells attenuate adipose inflammation and obesity through polarizing M2 macrophages and beiging in white adipose tissue. Diabetes. (2018) 67:235–47. 10.2337/db17-0356 29133512

[31] Nagy L Szanto A Szatmari I Széles L. Nuclear hormone receptors enable macrophages and dendritic cells to sense their lipid environment and shape their immune response. Physiol Rev. (2012) 92:739–89. 10.1152/physrev.00004.2011 22535896

[32] Moore KJ Rosen ED Fitzgerald ML Randow F Andersson LP Altshuler D. The role of PPAR-gamma in macrophage differentiation and cholesterol uptake. Nat Med. (2001) 7:41–7. 10.1038/83328 11135614

[33] Wang H Yang Y Sun X Tian F Guo S Wang W. Sonodynamic therapy-induced foam cells apoptosis activates the phagocytic PPARγ-LXRα-ABCA1/ABCG1 pathway and promotes cholesterol efflux in advanced plaque. Theranostics. (2018) 8:4969–84. 10.7150/thno.26193 30429880 PMC6217053

[34] Claudel T Leibowitz MD Fiévet C Tailleux A Wagner B Repa JJ. Reduction of atherosclerosis in apolipoprotein E knockout mice by activation of the retinoid X receptor. Proc Natl Acad Sci USA. (2001) 98:2610–5. 10.1073/pnas.041609298 11226287 PMC30186

[35] Ruan XZ Moorhead JF Fernando R Wheeler DC Powis SH Varghese Z. PPAR agonists protect mesangial cells from interleukin 1beta-induced intracellular lipid accumulation by activating the ABCA1 cholesterol efflux pathway. J Am Soc Nephrol. (2003) 14:593–600. 10.1097/01.ASN.0000050414.52908.DA 12595494

[36] Li AC Binder CJ Gutierrez A Brown KK Plotkin CR Pattison JW. Differential inhibition of macrophage foam-cell formation and atherosclerosis in mice by PPARalpha, beta/delta, and gamma. J Clin Invest. (2004) 114:1564–76. 10.1172/JCI18730 15578089 PMC529277

